# Supplementary material for: Black phosphorus boosts wet-tissue adhesion of composite patches by enhancing water absorption and mechanical properties
Source: Nat Commun. 2024 Feb 22;15:1618. doi: 10.1038/s41467-024-46003-6 (PMC10883952; doi:10.1038/s41467-024-46003-6)
Supplement: Supplementary file 1 — Supplementary Information [file 41467_2024_46003_MOESM1_ESM.pdf]

Supplementary Information for

Black Phosphorus boosts wet-tissue adhesion of composite patches by enhancing water absorption and mechanical properties

Yuanchi Zhang[1], Cairong Li[1], Along Guo[1], Yipei Yang[2], Yangyi Nie[1], Jiaxin Liao[1], Ben Liu[1], Yanmei Zhou[1], Long Li[1], Zhitong Chen[3], Wei Zhang[1], Ling Qin[1, 5], Yuxiao Lai[1, 4, 6]*

[1] Centre for Translational Medicine Research & Development, Shenzhen Institute of Advanced Technology, Chinese Academy of Sciences, Shenzhen, China.

[2] Department of Orthopedic Surgery, Shenzhen Hospital, Southern Medical University, Shenzhen, China.

[3] Institute of Biomedical and Health Engineering, Shenzhen Institute of Advanced Technology, Chinese Academy of Sciences, Shenzhen, China.

[4] Guangdong Province Engineering Laboratory for Biomedical Materials Additive Manufacturing, Shenzhen, China.

[5] Musculoskeletal Research Laboratory, Department of Orthopaedics & Traumatology, The Chinese University of Hong Kong, Hong Kong SAR, China.

[6] The Key Laboratory of Biomedical Imaging Science and System, Chinese Academy of Sciences, Shenzhen, China.

*Corresponding author

E-mail: yx.lai@siat.ac.cn (YX. Lai)

Supplementary Methods

Preparation of HAMA and PAA-DA

The MA modified HA (HAMA) is a semisynthetic photocrosslinkable hydrogel with excellent biocompatibility and modifiability, which is popular in biomedical engineering applications[1]. HA (2g) was first solved in the deionized water and dimethyl formamide (DMF, Aladdin Bio-Chem Technology, Shanghai, China). MA (10 ml) was added to the HA solvent with stirring where the pH value was adjusted to 8-9. The reaction was continued to stir overnight at 4°C. Then preliminary HAMA products were dialyzed against distilled water for 3 days and lyophilized to obtain the purified HAMA products. HAMA was self-crosslinked under the UV light (365 nm, ~68 mW cm^{-2}) and crosslinked with Gel in presence of the EDC/NHS subsequently (Supplementary Fig. 1a and b). PAA (4.32 g), EDC (2.88 g), NHS (1.73 g) and DA (2.84 g) were added into the buffer solution (pH<7) with stirring for 24 h under the protection of nitrogen. After that, the solution was freeze-dried to remove solvent using a vacuum lyophilizer for 48h (Bo Yi Kang FD-1-50, China) to obtain the dried product of the dopamine modified polyacrylic acid (PAA-DA) (Supplementary Fig. 1c).

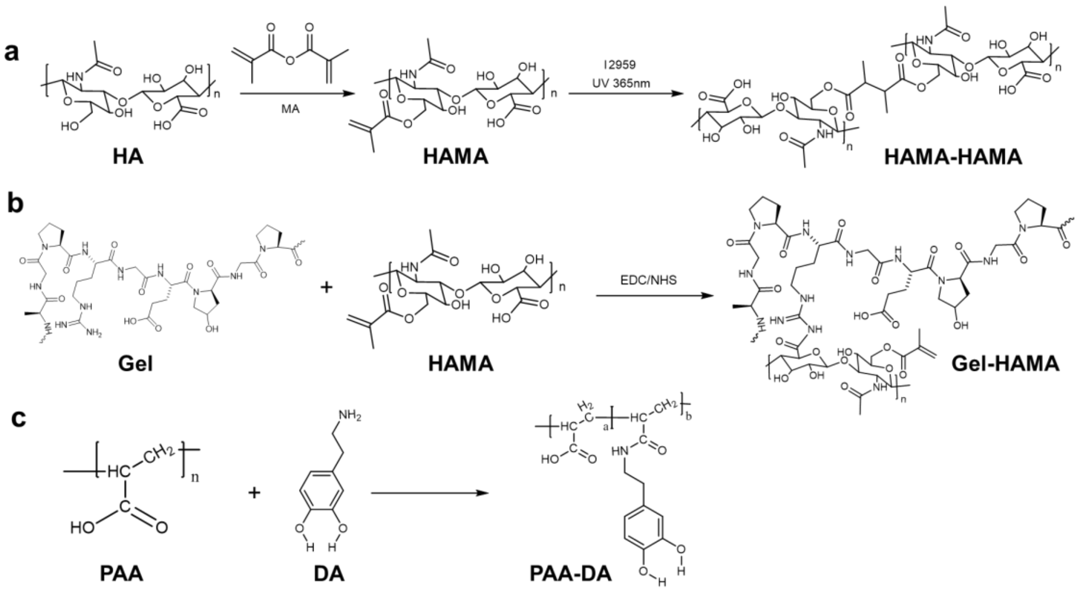

Supplementary Fig. 1 Preparation process of **(a)** HAMA, **(b)** Gel-HAMA and **(c)** PAA-DA**.**

The ^1H NMR spectra of HAMA and PAA-DA were analyzed to investigate the degree of methacrylation of HA and the grafting ratio of DA onto PAA, respectively. The sample was dissolved in D_2O in an NMR tube, and ^1H NMR measurement was carried out using an NMR spectrometer (Bruker Ascend 400M, Bruker, China). BP nanosheets were observed by SEM (ZEISS SUPRA® 55, Carl Zeiss, Germany) at an accelerating voltage of 10 kV and the transmission electron microscopy (TEM, JEM–3200FS, Beijing, China) at an acceleration voltage of 200 kV.

The graft-ratio of MA on pure HA calculated from ^1H NMR spectra was ~20% (Supplementary Fig. 2a). PAA-DA was prepared in advance, where the graft-ratio of DA was ~68% (Supplementary Fig. 2b). The SEM and TEM images of BP nanosheets showed that BP had a sheet size of ~100 nm – 1 μm (Supplementary Fig. 2c and 2d).

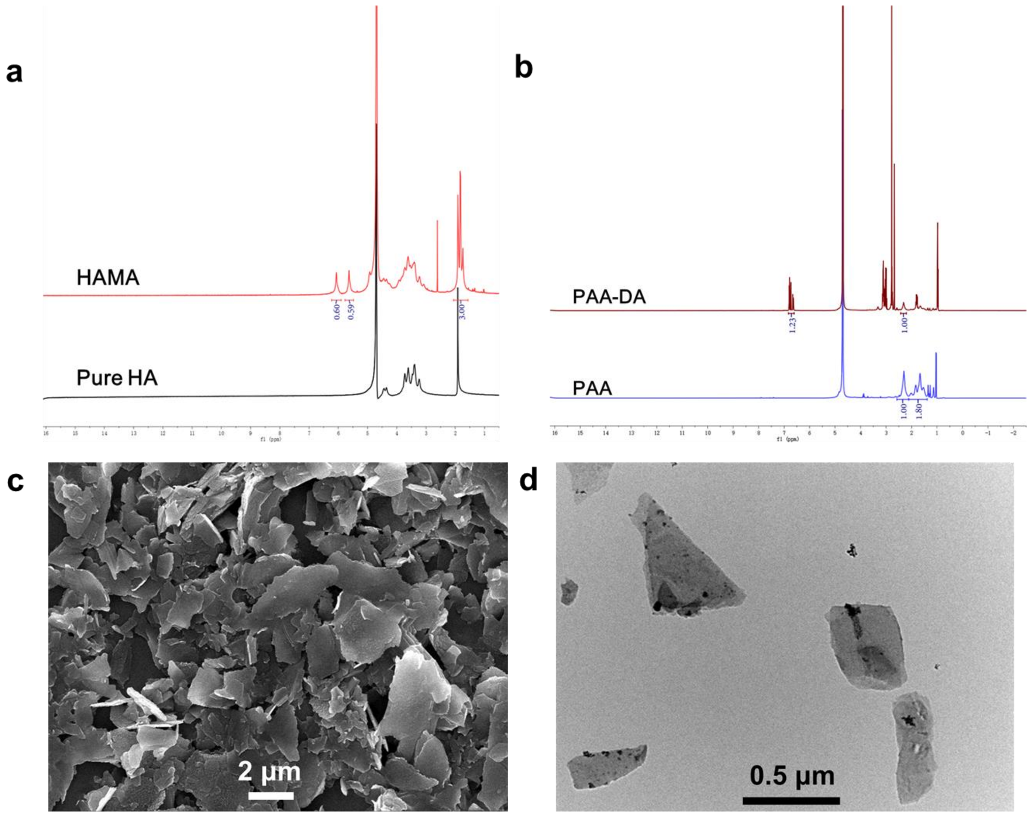

Supplementary Fig. 2 ^1H NMR (400 MHz, D_2O) spectra of (a) MA-grafted HA and **(b)** DA-grafted PAA. **(c)** SEM and **(d)** TEM images of the BP nanosheets. The images were repeated at least twice with consistent results.

Preparation of the CPB

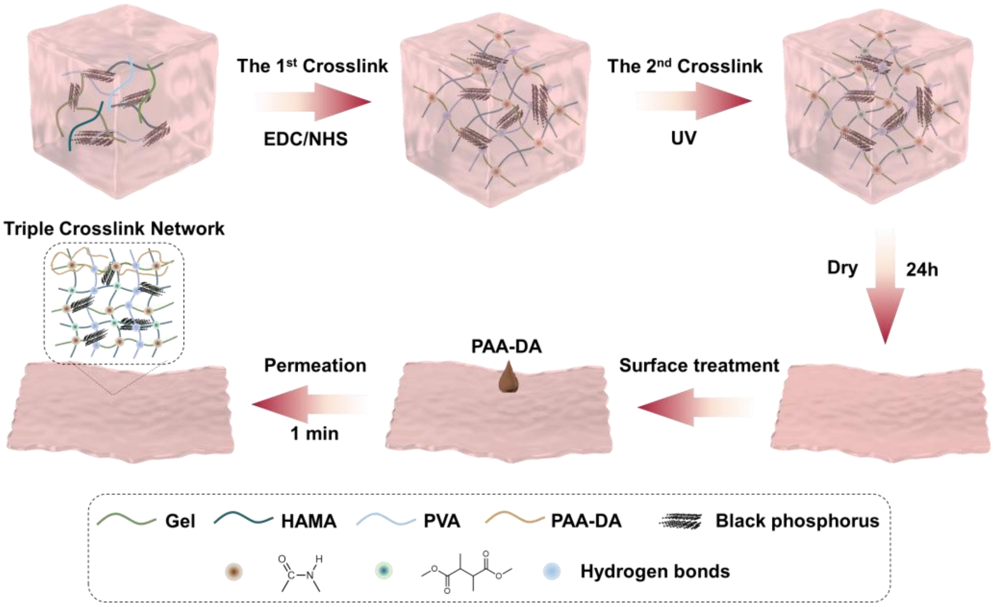

Supplementary Fig. 3 Schematic illustration of preparation of CPB. HAMA, PVA, BP, EDC, NHS and I2959 were mixed first. Gel solution was added into the mixture with fast stirring followed by being poured into a glass mold. The inter-crosslink bonds between HAMA and Gel formed due to the presence of the EDC/NHS linker. Subsequently, the mixture was placed under the UV light, resulting in the self-crosslink of HAMA. The hydroxyl groups of PVA could formed hydrogen bonds (HBs) with the functional groups such as NH- groups of HAMA and Gel to further enhance mechanical performance. The initial CPB product was obtained after the sample being dried overnight at room temperature (RT). The PAA-DA was dropwise added on the surface of the dried film to form the topological entanglement. Finally, CPB with triple crosslink network was prepared.

Characterizations of CP and CPB

The morphological characterization of the lyophilized CP and CPB was conducted by the scanning electron microscope with Energy Dispersive Spectrometer (SEM-EDS) (ZEISS SUPRA® 55, Carl Zeiss, Germany) at an accelerating voltage of 5 kV. CPB was directly adhered to harvest nude mice skin to evaluate its contact and flexibility. Fourier transform infrared spectrophotometer (FTIR) and Raman spectra were performed to confirm the introduction of BP nanosheets by a FTIR spectrometer (Thermo Nicolet iS5, USA) and a Raman Microscope equipment (Thermo Fisher DXR2 xi, USA) with 532 nm laser excitation at RT, respectively. XPS was performed to analyze the protonated and deprotonated forms of the amine-containing and phosphorus-containing chemicals in CPB by a XPS spectrometer (Thermo Scientific Nexsa, USA), using an Al Kα (λ = 0.83 nm, $h\upsilon$ = 1486.6 eV) X-ray source operated at 72 W.

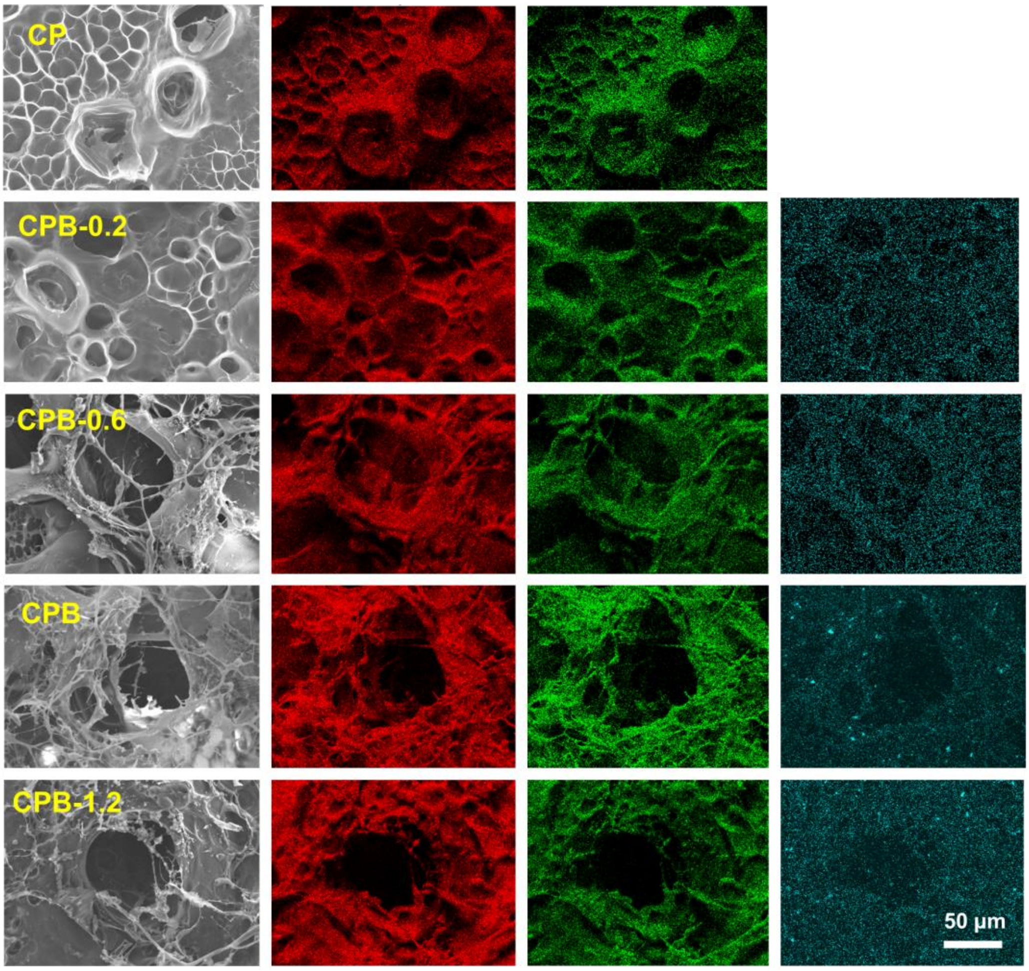

Supplementary Fig. 4 SEM and EDS images of CP and CPB with various contents

of BP nanosheets: CP: 0 mg, CPB-0.2: 0.2 mg, CPB-0.6: 0.6 mg, CPB: 1 mg, CPB-1.2: 1.2 mg. Red: C; Green: O; Blue: P. The micrographs were repeated at least twice with consistent results.

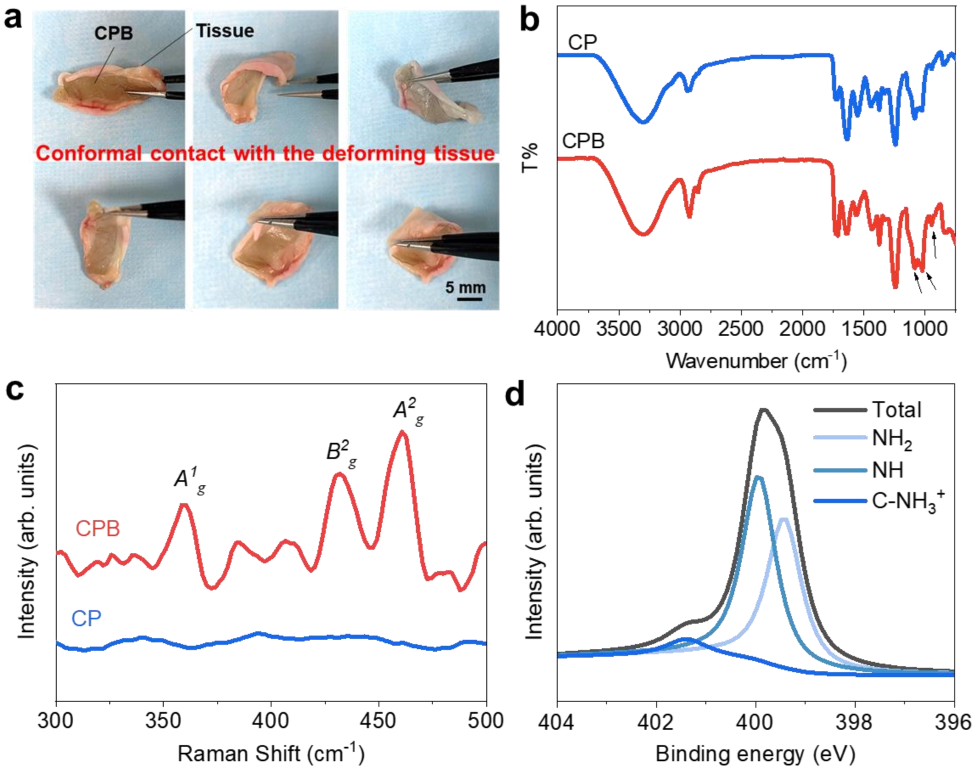

Supplementary Fig. 5 Characterizations of CP and CPB. **(a)** Flexibility. **(b)** FTIR. Black arrow from left to right: the characteristic bands at 1086, 1024 and 947 cm^{-1}, respectively. **(c)** Raman spectra. CPB: A^1_g at ~360.4 cm^{-1}, B^2_g at ~431.8 cm^{-1}, A^2_g at ~460.7 cm^{-1}. **(d)** The nitrogen binding energies (N1*s*) in the XPS spectra of CP.

Density functional theory (DFT) calculations

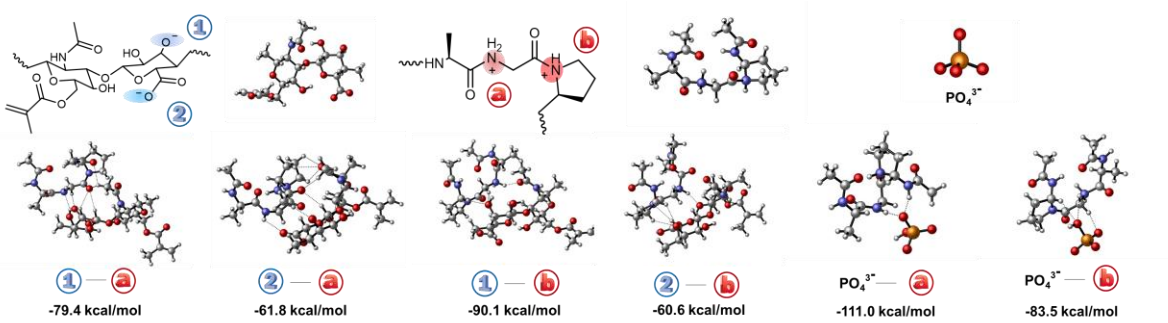

Supplementary Fig. 6 DFT calculations. Simulated structure and binding energy of HAMA ① – Gel (a), HAMA ① – Gel (b), HAMA ② – Gel (a), HAMA ② – Gel (b), and PO_4^{3-}-Gel (a, b) pairs.

Mechanical properties

The tensile tests were carried out by using an Instron tester (Instron Electropuls E10000, USA) at a testing rate of 1 mm min^{-1} at RT. Young's Modulus, stress strength and elongation at break were derived from the stress-strain curves for comparison (Supplementary Fig. 7). Most tissue adhesives have a low strength at dry state, leading to further decreased mechanical and adhesive performance at wet state. The strength of these patches was highly enhanced due to the triple network compared with a great many adhesive hydrogels[2, 3, 4]. In addition, the mechanical properties of CPB with various ratios of components were investigated. With higher ratios of PVA or higher graft-rations of HAMA, the Young's modulus and tensile strength were improved while the elongation at break was decreased. CPB with higher ratios of BP nanosheets had improved Young's modulus, tensile strength and elongation at break. Therefore, CPB had enough stiffness and flexibility to maintain the position and ensure satisfactory deformation during tissue movement in a wet condition.

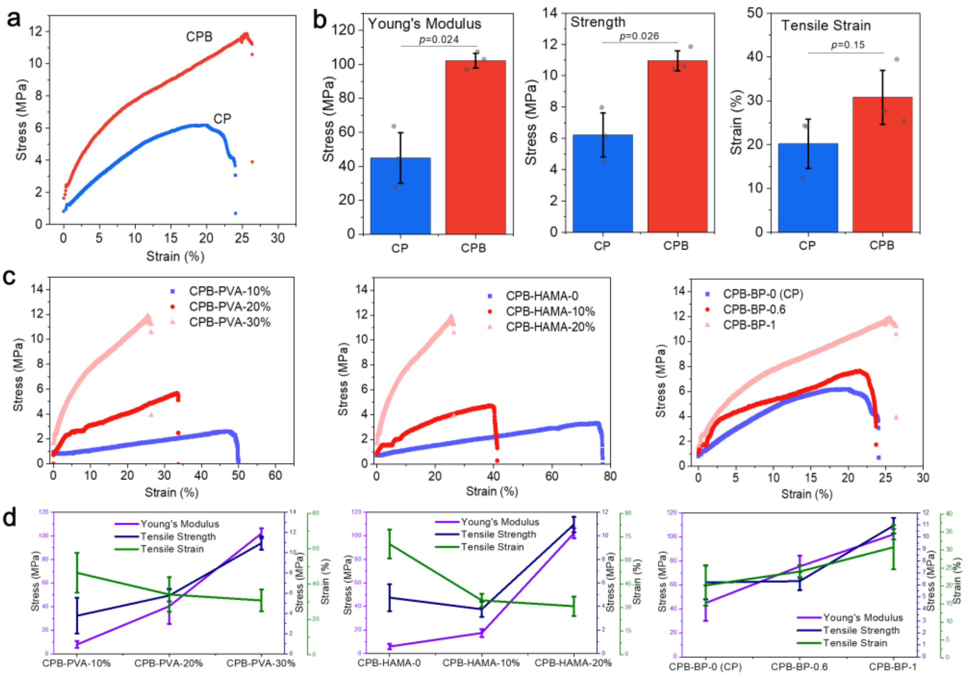

Supplementary Fig. 7 Mechanical properties. (a) Stress-stain curves of CP and CPB. **(b)** Young's modulus, tensile strength and elongation at break derived from (a). **(c)** Stress-stain curves of CPB with various ratios of PVA (left), various graft-ratios of HAMA (middle), various ratios of BP nanosheets (right), respectively. **(d)** Mechanical data derived from (c), respectively. Values in (**b**) and (**d**) represent the mean and standard deviation ($n = 3$ independent samples). Statistical analyses were performed by using two-tailed Student's *t*-test. No adjustments were made for multiple comparisons. *P* values less than 0.05 were considered statistically significant differences between the compared groups.

Electrical resistivity

The electrical resistivity of the patches with the same size of 10 × 10 × 0.2 mm were characterized using an Agilent 34411A Digital Multimeter, and the measured data were collected using the LabVIEW software (National Instrument, USA). As presented in Supplementary Fig. 8, the electrical resistivity of materials noticeably decreased by incorporating BP nanosheets, and even reached the similar value of Cu foil. Among them, the CPB showed the optimum value.

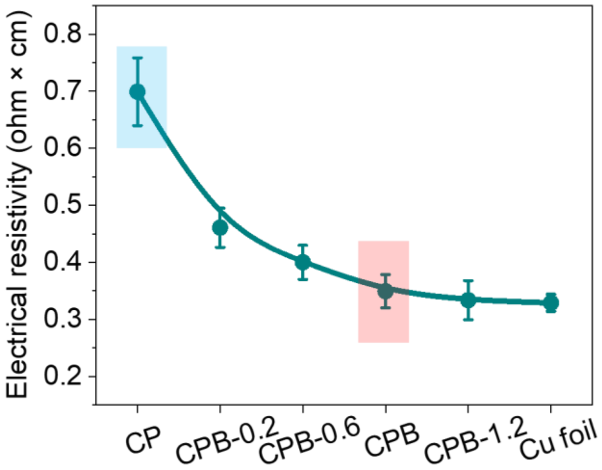

Supplementary Fig. 8 Electrical resistivity of CP and CPB with various contents of BP nanosheets: CP: 0 mg, CPB-0.2: 0.2 mg, CPB-0.6: 0.6 mg, CPB: 1 mg, CPB-1.2: 1.2 mg. Values represent the mean and standard deviation ($n = 3$ independent samples).

Degradation *in vitro*

The degradation of CP and CPB was evaluated by placing the patches in a phosphate-buffered saline (PBS) solution (pH = 7.4) with a ratio of 0.1g mL^{-1} according on ISO 10993-12, followed by putting in a thermostatic water bath bed (70 rpm, 37 ℃). The specimens were taken out and dried in a vacuum oven at RT for 48 h every week before weighting the samples.

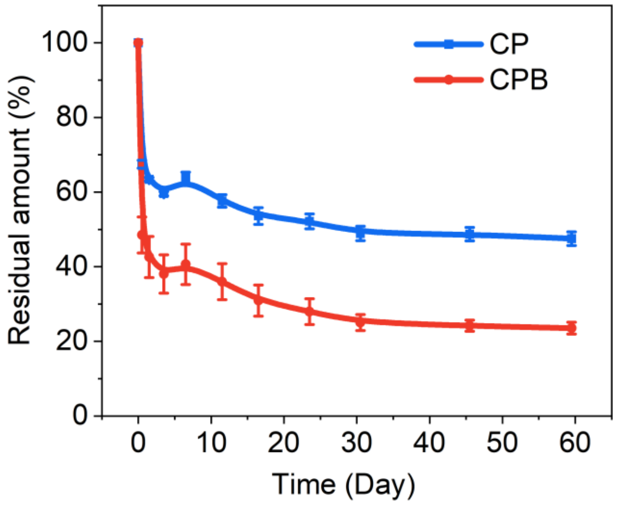

Supplementary Fig. 9 Degradation *in vitro* of CP and CPB in 60 days. Values represent the mean and standard deviation ($n = 3$ independent samples).

Photothermal performance

The photothermal effect of CP and CPB was investigated at dry state and wet state by using a NIR laser in air (808nm, P=1 W cm^{-2}, LWIRPD-10F, Laserwave, Beijing, China). The data of temperature changes were recorded by a NIR camera (FLIR One, FLIR Systems, Inc., Hong Kong, China). Because there was no BP nanosheets or other photothermal agents, the CP had no temperature increase under NIR light at dry state and wet state (Supplementary Fig. 10). In contrast, the temperature of CPB had a high ramp rate during first 60 seconds, where increased from room temperature to ~51℃ at dry state and to ~50°C at wet state, respectively. For further evaluating the photothermal stability of CPB at wet state, the temperature change of CPB under NIR light for 60 seconds (laser on) was recorded, followed by natural cooling to room temperature after the NIR laser was turned off (laser off). Even the cycles repeated 5 times, the efficiency of the photothermal effects had little decay, proving the excellent photothermal stability of CPB.

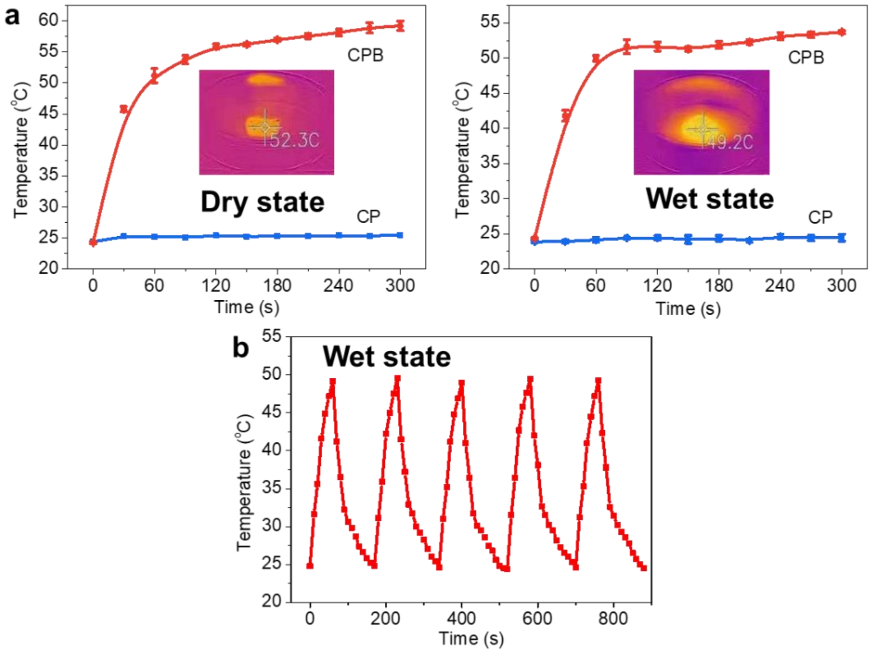

Supplementary Fig. 10 Photothermal effect. (a) Photothermal heating curves of CP and CPB at dry state and wet state irradiated by the NIR laser (808 nm, 1 W cm^{-2}). Insert: infrared thermal images of CPB at 60 seconds. **(b)** Temperature elevation of CPB for five laser on/off cycles (808 nm, 1 W cm^{-2}) at wet state. Values in (**a**) represent the mean and standard deviation ($n = 3$ independent samples).

Adhesive performance

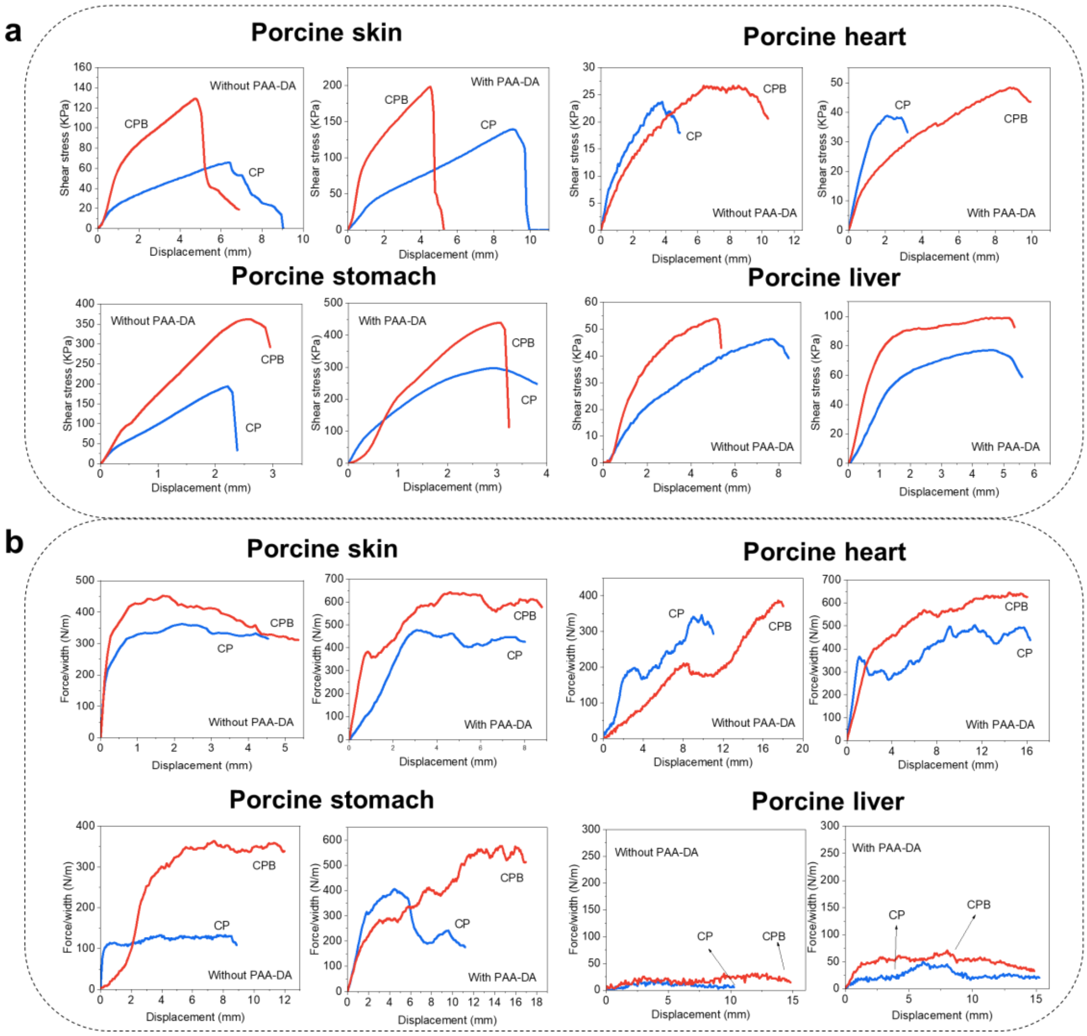

Supplementary Fig. 11 Adhesive performance. (a) Lap-shear adhesive performance of CP and CPB adhered to wet porcine tissues from various organs (skin, heart, stomach, liver). **(b)** Modified 180 ° peel adhesive performance of CP and CPB adhered to wet porcine tissues from various organs (skin, heart, stomach, liver).

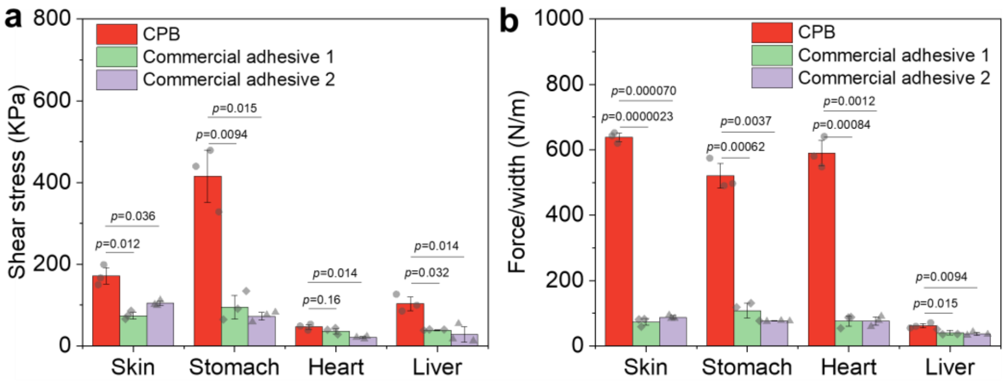

Supplementary Fig. 12 Adhesive performance of CPB and the commercial tissue adhesives adhered to wet porcine tissues from various organs (skin, heart, stomach, liver). **(a)** Shear stress. **(b)** Interfacial toughness. Values in (**a**) and (**b**) represent the mean and standard deviation ($n = 3$ independent samples). Statistical analyses were performed by using two-tailed Student's *t*-test. No adjustments were made for multiple comparisons. *P* values less than 0.05 were considered statistically significant differences between the compared groups.

In addition, the adhesive performance of the patches adhered to dry porcine skin after 12 h were also evaluated. The shear stress of CP and CPB in the dry tissues group was ~1 KPa, which had no much difference (Supplementary Fig. 13a). In the wet tissues group, the shear stress of CPB (~79 KPa) was obviously higher compared with CP (~60 KPa) (Supplementary Fig. 13b). The stress difference between the dry and wet tissues groups should be attributed to water content of the patches[5]. The experimental results indicated that the enhanced water absorption capacity contributed more in wet adhesion of CPB than the increased mechanical property of CPB.

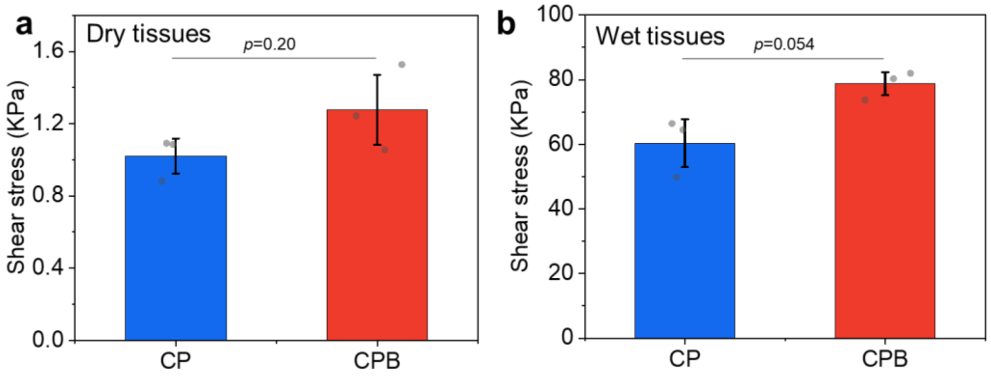

Supplementary Fig. 13 Adhesive performance of CP and CPB to dry porcine skin (a) and wet porcine skin (b) after 12 h. Values in (**a**) and (**b**) represent the mean and standard deviation ($n = 3$ independent samples). Statistical analyses were performed by using two-tailed Student's *t*-test. No adjustments were made for multiple comparisons. *P* values less than 0.05 were considered statistically significant differences between the compared groups.

***In vitro* cell studies**

Cell behaviors were investigated for evaluating the biological influences of various samples with or without NIR light. Sterilized CP and CPB were immersed in a minimum essential medium α (α-MEM, Gibco, USA) supplemented with 10% (v v^{-1}) fetal bovine serum (FBS, Gibco, USA) and 1% (v v^{-1}) penicillin/streptomycin (Gibco, USA) for 24 h at 37℃ in 5% CO_2 atmosphere for extraction according to ISO 10993-12. L929 cells (CCL-1, ATCC, Manassas, VA, USA) were cultured using the extract liquid (diluted 50 times) with the samples in a 96-well culture plate with a density of 5×10^3 cells $well^{-1}$ in an incubator under 37 ℃ and 5% CO_2. The Cell Counting Kit-8 (CCK-8, Dojindo, Japan) assay was conducted after being incubated for 1, 3, 5 and 7 days to determine the cells proliferation according to the manufacturer's instructions and literature[6, 7]. In addition, MCF-7 cells (HTB-22, ATCC, Manassas, VA, USA) were cultured using the extract liquid (diluted 50 times) in a 24-well culture plate with a density of 2×10^4 cells $well^{-1}$ in an incubator under 37 ℃ and 5% CO_2 for 24 h. Then CP and CPB were respectively put at the center of the wells followed by being irradiated by NIR light (808 nm, P=1 W cm^{-2}) for 5 minutes. After 6 h incubation, Live/Dead staining assay was carried out to determine the effects of CP and CPB on MCF-7 cells. Moreover, the Annexin V-FITC/ propidium iodide (PI) double-staining assay was also used to investigate cell death according to the protocol and literature[8]. The samples were detected using flow cytometry (BD FACSCanto II, USA).

At first, the Live/Dead staining results suggested the cells were normal in each group without NIR irradiation (Supplementary Fig. 14a). When being placed under NIR light for 5 minutes (808 nm, P=1 W cm^{-2}), the control group and CP group had no obvious change due to no photothermal (PT) effect. On the contrary, most tumour cells in the CPB group were killed due to the PT effect. In addition, the Annexin V-FITC/PI staining results proved that the majority (>95%) of the MCF-7 cell death on the CPB with NIR irradiation group (Supplementary Fig. 14b). These results suggested the potential of CPB for *in situ* photothermal treatment (PTT). In addition, NIR light was also proved to be biosafe for normal cells. To determine the cytotoxicity of the patches, L929 cells were used to investigate the cell viability and proliferation. The control

group had no material treatment. The results indicated that the L929 cells proliferated more along with time in all groups (Supplementary Fig. 14c). There was no significant difference between the control group and the CPB group, as well as the CPB group and the CP group, which demonstrated the patches had good cell biocompatibility.

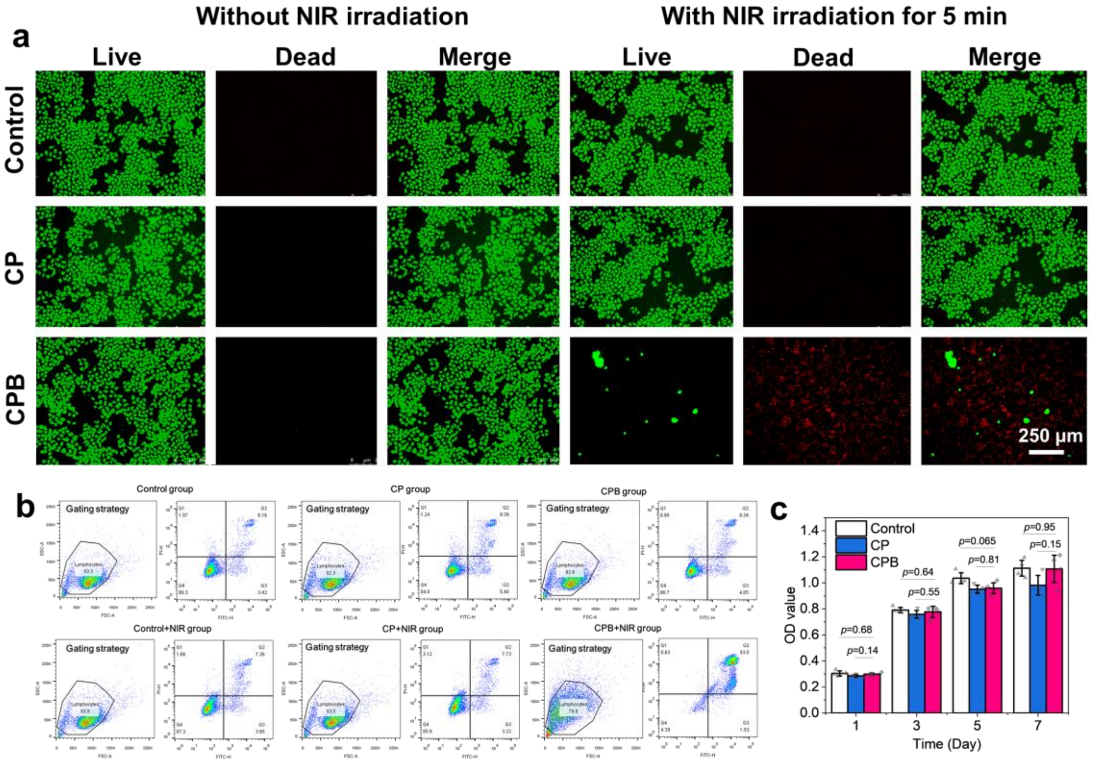

Supplementary Fig. 14 ***In vitro*** **cell studies. (a)** Fluorescence images of tumour cells after adding the patches without and with NIR irradiation (808 nm, P=1 W cm^{-2}) for 5 minutes. The live cells are in green and dead cells are in red. **(b)** Gating strategy (left) and representative Annexin V-FITC/PI scatter plots of MCF-7 cells after 6 h of treatment (right). **(c)** Cell proliferation of L929 cells. Values in (**c**) represent the mean and standard deviation ($n = 4$ independent samples). Statistical analyses were performed by using two-tailed Student’s *t*-test. No adjustments were made for multiple comparisons. *P* values less than 0.05 were considered statistically significant differences between the compared groups.

Potential application of hemostasis and activity monitor

CP and the commercial products (e.g. Gelatin Sponge and Gauze) were used as control groups for evaluating the hemostatic effect in a normal SD rat liver perforation wound model. A total of 15 SD rats (male, weight of 250-300 g, 7-8 weeks) were randomly divided into the CP group, Gelatin Sponge group, and Gauze group. Then the livers of the rats were lifted and placed on the surface of preweighted filter paper, and a circular perforation wound (diameter of 6 mm) was created for hemorrhage. The sample (diameter of ~10 mm) was weighted in advance. Next the corresponding sample in each control group was directly adhered to the bleeding site and the hemostatic process was recorded with a digital camera. The blood loss was calculated by determining the total weight of the blood absorbed by the filter paper and the sample, respectively. Similarly, the hemostatic effect of CP, the commercial Gelatin Sponge and Gauze were evaluated by a normal SD rat heart perforation wound model, where the hearts of rats were lifted and a circular perforation wound (diameter of 6 mm) was created for hemorrhage. The corresponding sample (diameter of ~10 mm) in each control group was immediately adhered to the bleeding sites, and the state was recorded with a digital camera. (Supplementary Fig. 15).

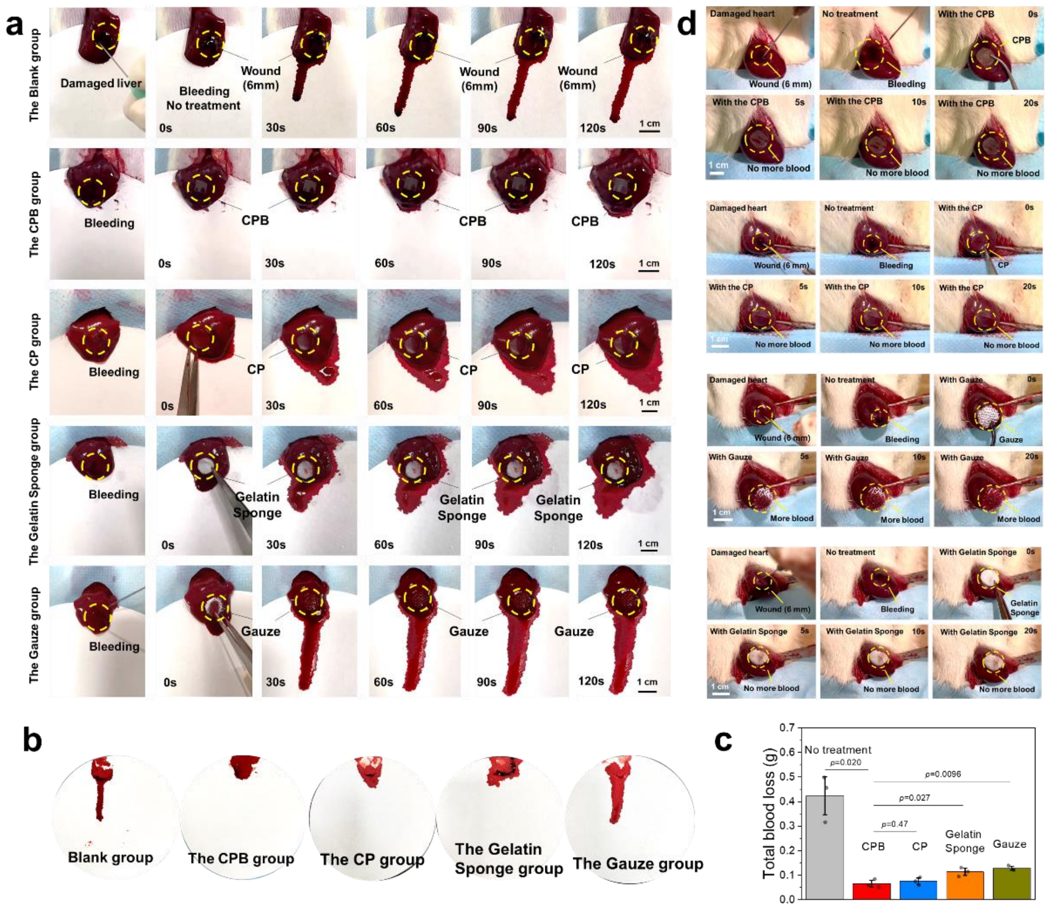

Supplementary Fig. 15 ***In vivo*** **hemostasis. (a)** Images of the hemostatic effect for the damaged liver in the blank group, CPB group, CP group, Gelatin Sponge group and Gauze group. Yellow dotted line: the position of the wound or the sample. **(b)** Bloodstain on the surface of the filter paper in the blank group, CPB group, CP group, Gelatin Sponge group and Gauze group at 120 seconds. **(c)** Total blood loss in the blank group, CPB group, CP group, Gelatin Sponge group and Gauze group. **(d)** Images of the hemostatic effect of the CP, Gelatin Sponge and Gauze in a rat dynamic heart perforation wound model. Yellow dotted line: the position of the wound or the sample. Values in (**c**) represent the mean and standard deviation ($n = 3$ independent samples). Statistical analyses were performed by using two-tailed Student's *t*-test. No adjustments were made for multiple comparisons. *P* values less than 0.05 were considered statistically significant differences between the compared groups.

Potential application of tumour recurrence prevention

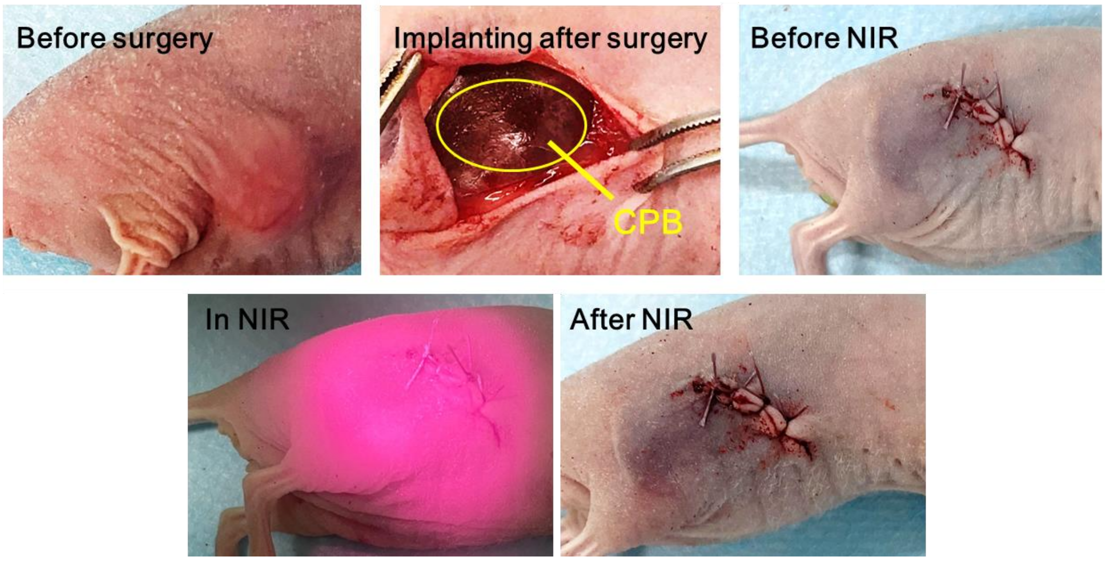

Supplementary Fig. 16 Process of tumour removal and CPB implantation with NIR light irradiation (808 nm, P=1 W/cm^2).

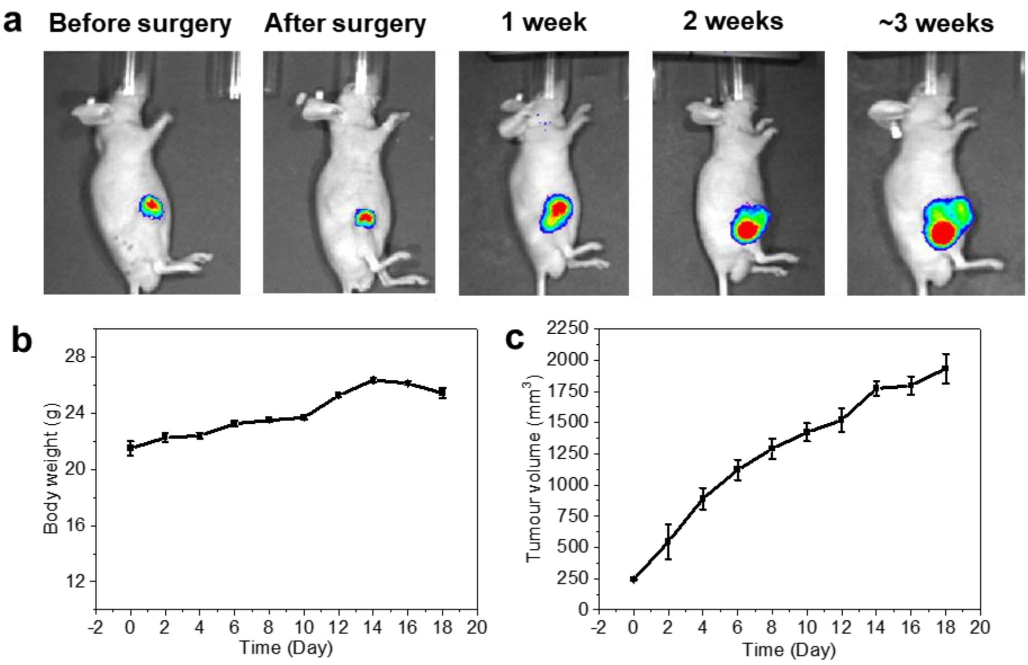

Supplementary Fig. 17 Tumour growth in blank group. (a) Fluorescence images of the tumour-bearing mice before and after the surgical time-point (no treatment in this blank group) immediately, 1week, 2 weeks and ~3 weeks. **(b)** Body weight changes of the nude mice recorded every two days. **(c)** Tumour volume changes recorded every two days. Values in (**b**) and (**c**) represent the mean and standard deviation ($n = 3$ independent samples).

Degradation *in vivo*

Total nine SD rats were divided into three groups and then anesthetized. Back hair of the rats was removed, and the skin was aseptically prepared. Subsequently, a small skin incision (~15 mm) was made on the back and 3 separated subcutaneous pockets were created for inserting the patches (*n*=3) per rat in each group. The skin was sutured at the incision site. After 2, 4 and 8 weeks implantation, the patches were collected from the rat and then dried in a vacuum oven at RT for 48 h. Residual amount was calculated by the equation:

$$\text{Residual amount} = \frac{W_1}{W_0} \times 100\% \qquad (1)$$

Where W_0 and W_1 represent the weight of patches before and after implantation, respectively.

After 8 weeks, CP had over 50% residual amount, and CPB without and with NIR irradiation could degrade to ~40-42% residual amount (Supplementary Fig. 18). The NIR light had no obvious effect on the biodegradation of the patches in this work. The high biodegradation rate of CPB might be attributed by the enhanced water absorbing capacity. The components of the patches were biocompatible and biodegradable, so the patches did not need extra surgery to remove.

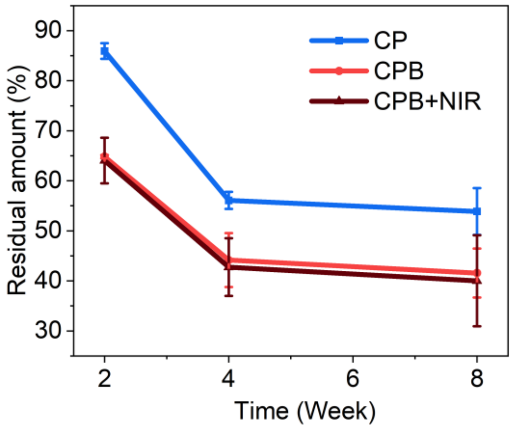

Supplementary Fig. 18 Residual amount of the patches after being implanted for 2, 4 and 8 weeks. CP group: implanting CP; CPB group: implanting CPB; CPB+NIR group: implanting CPB and irradiated by NIR light (808 nm, P=1 W cm^{-2}) for 5 minutes. Values represent the mean and standard deviation (*n* = 3 independent samples).

Histocompatibility

Total nine SD rats were randomly divided and prepared as described in the *Degradation in vivo* section. 3 separated subcutaneous pockets were created in one rat for inserting the CP, CPB and CPB with NIR light for 5 minutes (808 nm, 1 W cm^{-2}), respectively. The skin tissues without any treatment and implantation were collected as the control group. After 2, 4 and 8 weeks, H&E (Sigma-Aldrich, Shanghai, China) were used to stain the tissues at the implanting sites for histological evaluation. After being stained, the sections were observed by a light microscope (Olympus IX71, Japan). The experimental results in Supplementary Fig. 19 proved there was no obviously increased inflammatory cells around the patches at each time-point.

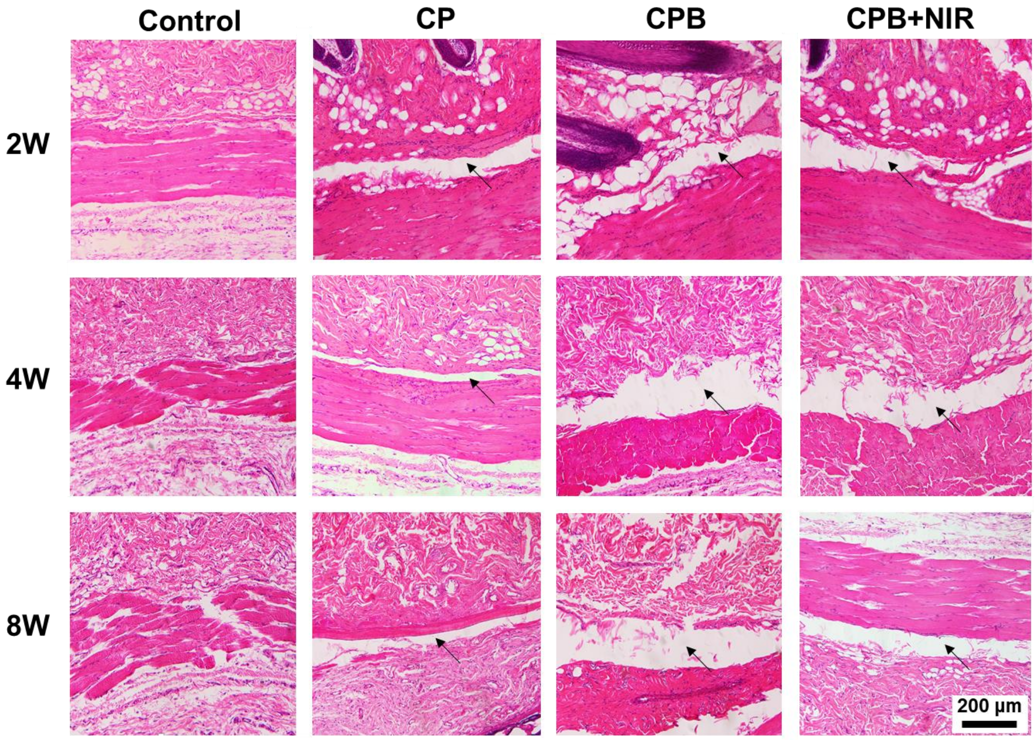

Supplementary Fig. 19 H&E-stained sections of skin tissues from the sacrificed rats in each group after 2, 4 and 8 weeks. CP group: implanting the CP; CPB group: implanting the CPB; CPB+NIR group: implanting CPB and irradiated by NIR light (808 nm, P=1 W cm^{-2}) for 5 minutes. Black arrow: the position of patches. The histological investigations were repeated at least twice for consistent results.

Supplementary References

1. Ouyang L, *et al.* Expanding and optimizing 3D bioprinting capabilities using complementary network bioinks. *Sci. Adv.* **6**, eabc5529 (2020).

2. Lin X, *et al.* A viscoelastic adhesive epicardial patch for treating myocardial infarction. *Nat. Biomed.* **3**, 632-643 (2019).

3. Luo J, *et al.* A Highly Stretchable, Real-Time Self-Healable Hydrogel Adhesive Matrix for Tissue Patches and Flexible Electronics. *Adv. Healthc. Mater.* **9**, 1901423 (2020).

4. Zhou L, *et al.* Injectable Self-Healing Natural Biopolymer-Based Hydrogel Adhesive with Thermoresponsive Reversible Adhesion for Minimally Invasive Surgery. *Adv. Funct. Mater.* **31**, 2007457 (2021).

5. Zhou Z, Lei J, Liu Z. Effect of water content on physical adhesion of polyacrylamide hydrogels. *Polymer* **246**, 124730 (2022).

6. Wang Y, *et al.* Bone remodeling-inspired dual delivery electrospun nanofibers for promoting bone regeneration. *Nanoscale* **11**, 60-71 (2019).

7. Li C, *et al.* Preparation and evaluation of osteogenic nano-MgO/PMMA bone cement for bone healing in a rat critical size calvarial defect. *J Mater Chem B* **8**, 4575-4586 (2020).

8. Ma L, *et al.* A novel photothermally controlled multifunctional scaffold for clinical treatment of osteosarcoma and tissue regeneration. *Mater. Today* **36**, 48-62 (2020).
