# Supplementary material for: Black phosphorus boosts wet-tissue adhesion of composite patches by enhancing water absorption and mechanical properties
Source: Nat Commun. 2024 Feb 22;15:1618. doi: 10.1038/s41467-024-46003-6 (PMC10883952; doi:10.1038/s41467-024-46003-6)
Supplement: Supplementary file 5 — Reporting Summary [file 41467_2024_46003_MOESM5_ESM.pdf]

nature portfolio

Corresponding author(s): Yuxiao Lai

Last updated by author(s): Jan 17, 2024

Reporting Summary

Nature Portfolio wishes to improve the reproducibility of the work that we publish. This form provides structure for consistency and transparency in reporting. For further information on Nature Portfolio policies, see our Editorial Policies and the Editorial Policy Checklist.

Statistics

For all statistical analyses, confirm that the following items are present in the figure legend, table legend, main text, or Methods section.

n/a	Confirmed	
☐	☒	The exact sample size (n) for each experimental group/condition, given as a discrete number and unit of measurement
☐	☒	A statement on whether measurements were taken from distinct samples or whether the same sample was measured repeatedly
☐	☒	The statistical test(s) used AND whether they are one- or two-sided<br>*Only common tests should be described solely by name; describe more complex techniques in the Methods section.*
☒	☐	A description of all covariates tested
☐	☒	A description of any assumptions or corrections, such as tests of normality and adjustment for multiple comparisons
☐	☒	A full description of the statistical parameters including central tendency (e.g. means) or other basic estimates (e.g. regression coefficient) AND variation (e.g. standard deviation) or associated estimates of uncertainty (e.g. confidence intervals)
☐	☒	For null hypothesis testing, the test statistic (e.g. F, t, r) with confidence intervals, effect sizes, degrees of freedom and P value noted<br>*Give P values as exact values whenever suitable.*
☒	☐	For Bayesian analysis, information on the choice of priors and Markov chain Monte Carlo settings
☒	☐	For hierarchical and complex designs, identification of the appropriate level for tests and full reporting of outcomes
☒	☐	Estimates of effect sizes (e.g. Cohen's d, Pearson's r), indicating how they were calculated

Our web collection on statistics for biologists contains articles on many of the points above.

Software and code

Policy information about availability of computer code

Data collection	Data were collected with various laboratory instruments and equipments. Such as: XPS spectrometer (Thermo Scientific Nexsa, USA), Contact Angle Meter (Jinhe JY-PHb, Chengd,e, China), NMR spectrometer (Bruker Ascend 400M, Bruker, China), TEM (JEM–3200FS, Beijing), Chinaconfocal fluorescence microscopy (ZEISS LSM 900, Germany) , scanning electron microscopy (ZEISS SUPRA® 55, Carl Zeiss, Germany), Instron tester (Instron Electropuls E10000, USA), Raman Microscope equipment (Thermo Fisher DXR2 xi, USA), MATLAB Platform (Mathworks, Natick, MA), NIR camera (FLIR One, FLIR Systems, Inc., Hong Kong, China) , flow cytometry (BD FACSCanto II, USA), in vivo imaging system (PerkinElmer, USA) and light microscope (Olympus IX71, Japan).
Data analysis	Origin 2022, CYLview 1.0 b, LabVIEW, Microsoft Excel 2013, SPSS 27, Gaussian 16. Revision C. 01.

For manuscripts utilizing custom algorithms or software that are central to the research but not yet described in published literature, software must be made available to editors and reviewers. We strongly encourage code deposition in a community repository (e.g. GitHub). See the Nature Portfolio guidelines for submitting code & software for further information.

Data

Policy information about availability of data

All manuscripts must include a data availability statement. This statement should provide the following information, where applicable:

- Accession codes, unique identifiers, or web links for publicly available datasets
- A description of any restrictions on data availability
- For clinical datasets or third party data, please ensure that the statement adheres to our policy

All data are available in the main text and Supplementary Information. Source data are provided with this paper. The data generated in this study have been deposited in the figshare database under the accession code https://doi.org/10.6084/m9.figshare.24548476.

Research involving human participants, their data, or biological material

Policy information about studies with human participants or human data. See also policy information about sex, gender (identity/presentation), and sexual orientation and race, ethnicity and racism.

Reporting on sex and gender	N/A
Reporting on race, ethnicity, or other socially relevant groupings	N/A
Population characteristics	N/A
Recruitment	N/A
Ethics oversight	N/A

Note that full information on the approval of the study protocol must also be provided in the manuscript.

Field-specific reporting

Please select the one below that is the best fit for your research. If you are not sure, read the appropriate sections before making your selection.

☒ Life sciences ☐ Behavioural & social sciences ☐ Ecological, evolutionary & environmental sciences

For a reference copy of the document with all sections, see nature.com/documents/nr-reporting-summary-flat.pdf

Life sciences study design

All studies must disclose on these points even when the disclosure is negative.

Sample size	Sample sizes were chosen on the basis of published literature on similar evaluations (for example, Nat Commun 12, 7156 (2021)). For each experiment, we have adopted at least n=3 biological replicates to calculate the statistical value of each analysis and the exact sample sizes and statistical data for each experiment are reported in the figure legends.
Data exclusions	No data were excluded from the analyses.
Replication	All experiments were repeated at least three times with reproducibility, and all attempts at replication were successful.
Randomization	All the tests were performed with randomly allocated experimental groups.
Blinding	All measurements were conducted in a blinded fashion.

Reporting for specific materials, systems and methods

We require information from authors about some types of materials, experimental systems and methods used in many studies. Here, indicate whether each material, system or method listed is relevant to your study. If you are not sure if a list item applies to your research, read the appropriate section before selecting a response.

Materials & experimental systems

n/a	Involved in the study
☒	☐ Antibodies
☐	☒ Eukaryotic cell lines
☒	☐ Palaeontology and archaeology
☐	☒ Animals and other organisms
☒	☐ Clinical data
☒	☐ Dual use research of concern
☒	☐ Plants

Methods

n/a	Involved in the study
☒	☐ ChIP-seq
☐	☒ Flow cytometry
☒	☐ MRI-based neuroimaging

Eukaryotic cell lines

Policy information about cell lines and Sex and Gender in Research

Cell line source(s)	L929 cells (ATCC, Manassas, VA, USA), MCF-7 cells (ATCC, Manassas, VA, USA)
Authentication	Authentication was performed by analysis of morphology.
Mycoplasma contamination	All cell lines tested negative for mycoplasma contamination.
Commonly misidentified lines (See ICLAC register)	No commonly misidentified lines were used in this study.

Animals and other research organisms

Policy information about studies involving animals; ARRIVE guidelines recommended for reporting animal research, and Sex and Gender in Research

Laboratory animals	5~6-weeks-old female Balb/c nude mice and 7~8-week-old male SD rats were purchased from were purchased from Beijing Vital River Company, China.
Wild animals	The study did not involve wild animals.
Reporting on sex	The findings of the experiments apply to both females and males.
Field-collected samples	This study did not involve samples collected from the field.
Ethics oversight	All animal experiments were approved by the Institutional Animal Care and Use Committee (IACUC), Shenzhen Institutes of Advanced Technology (SIAT), Chinese Academy of Sciences (SIAT-IACUC-211010-YGS-ZYC-A2067) and performed following the guidelines published by the NIH.

Note that full information on the approval of the study protocol must also be provided in the manuscript.

Flow Cytometry

Plots

Confirm that:

☒ The axis labels state the marker and fluorochrome used (e.g. CD4-FITC).

☒ The axis scales are clearly visible. Include numbers along axes only for bottom left plot of group (a 'group' is an analysis of identical markers).

☒ All plots are contour plots with outliers or pseudocolor plots.

☒ A numerical value for number of cells or percentage (with statistics) is provided.

Methodology

Sample preparation	MCF-7 cells (ATCC, Manassas, VA, USA) were cultured using the extract liquid (diluted 50 times) in a 24-well culture plate with a density of 2×10000 cells well-1 in an incubator under 37 °C and 5% CO2 for 24h. Then the CP and CPB were respectively put at the center of the wells followed by being irradiated by NIR light (808 nm, P=1 W cm-2) for 5 min. After 6 hours incubation, the cells were stained with the annexin V/PI dead cell apoptosis kit (Invitrogen, V13241), and analyzed by flow cytometry (BD FACSCanto II, USA)
Instrument	flow cytometry (BD FACSCanto II, USA)
Software	FlowJo _V10 (FlowJo, LLC)

Cell population abundance	*Describe the abundance of the relevant cell populations within post-sort fractions, providing details on the purity of the samples and how it was determined.*
Gating strategy	*Describe the gating strategy used for all relevant experiments, specifying the preliminary FSC/SSC gates of the starting cell population, indicating where boundaries between "positive" and "negative" staining cell populations are defined.*

☒ Tick this box to confirm that a figure exemplifying the gating strategy is provided in the Supplementary Information.
