# Supplementary material for: Black phosphorus boosts wet-tissue adhesion of composite patches by enhancing water absorption and mechanical properties
Source: Nat Commun. 2024 Feb 22;15:1618. doi: 10.1038/s41467-024-46003-6 (PMC10883952; doi:10.1038/s41467-024-46003-6)
Supplement: Supplementary file 7 — Description of Additional Supplementary Files [file 41467_2024_46003_MOESM7_ESM.pdf]

Title: Supplementary Movie 1.
Description: Adhesive performance – skin

Title: Supplementary Movie 2.
Description: Adhesive performance – sealing

Title: Supplementary Movie 3.
Description: Hemostatic effect of CPB
